# Duration of stunting impacts compensatory growth and carcass quality of farmed milkfish, *Chanos chanos* (Forsskal, 1775) under field conditions

**DOI:** 10.1038/s41598-019-53092-7

**Published:** 2019-11-14

**Authors:** Somu Sunder Lingam, Paramita Banerjee Sawant, Narinder Kumar Chadha, Kurcheti Pani Prasad, A. P. Muralidhar, Karthireddy Syamala, K. A. Martin Xavier

**Affiliations:** 1 Division of Aquaculture, ICAR- Central Institute of Fisheries Education, Versova, Mumbai, 400061 India; 2 Division of Aquatic Animal Health Management, ICAR- Central Institute of Fisheries Education, Versova, Mumbai, 400061 India; 3 Kakinada Centre, ICAR- Central Institute of Fisheries Education, Kakinada, Andhra Pradesh 533001 India; 4 Division of Post-Harvest Technology, ICAR- Central Institute of Fisheries Education, Versova, Mumbai, 400061 India

**Keywords:** Zoology, Animal physiology

## Abstract

An 18-months field trial was performed to explore the effect of duration of stunting on growth, digestive enzymes and carcass quality in *Chanos chanos*. Milkfish fry (weight of 1.25 ± 0.03 g and length of 5.53 ± 0.03 cm) were stocked in earthen ponds of 0.02 ha, in triplicate, for different duration of stunting, viz., 4 months (Treatment-1; T4), 8 months (Treatment-2; T8) and 12 months (Treatment-3; T12) and a normal seed (Control; C) separately. In the stunting phase, fish were stocked at higher stocking density (0.2 million/ha) and fed de-oiled rice bran at sub-optimal level. Post-stunting or re-feeding phase commenced immediately after completion of respective stunting duration and fish were reared for the rest of the period to complete the total rearing period of 18 months. In post-stunting, fish stocking density was adjusted to (5000 pieces/ha) and fed at an optimum level (3%). At the end of stunting phase, the study found a significant reduction in growth, survival, digestive enzymes activity, except protease in the T4 group, and carcass nutrients composition of stunted fish. However, in the initial phase of post-stunting, T8 group exhibited an elevated specific growth rate (5.00 ± 0.092%/day), body weight gain (80.82 ± 1.28 g), amylase (0.585 ± 0.021 U/mg protein), protease (5.48 ± 0.13 U/mg protein), and lipase activity (7.92 ± 0.32 U/mg protein). All stunted fish groups displayed a compensatory growth response in post-stunting, but a complete growth compensation was observed in T8 group, which resulted in better feed conversion ratio (3.03 ± 0.04) feed efficiency ratio (0.33 ± 0.01), protein efficiency ratio (1.91 ± 0.03), survival (91.38 ± 0.07%) and digestive enzyme activities. Similarly, at the end of post-stunting, carcass analysis revealed a complete restoration of nutrients in stunted fish and significantly higher protein content in T8 group. Further, the study found lower meat and higher bone contents in normally reared fish than the post-stunted fish which revealed the carcass quality improvement in post-stunted fish thus indicates superiority of the stunting process over normal rearing. Overall, the study suggests that stunting of milkfish, for 8 months (T8), positively affects its growth, survival, digestive enzyme activities and carcass quality which in turn, shall help to overcome the contemporary challenges in milkfish culture.

## Introduction

Aquaculture, the underwater agriculture, is fulfilling half of the animal protein requirement of the world population through its protein-rich product, fish^1^. In the present global scenario, increasing population and limited natural resources, rearing of fish in captive conditions is considered as a most efficient and cheapest animal protein production system compared to other animal protein production sectors^2^. However, in the evolution context of intensive modern fish culture practices, feed utilization efficacy of fish is considered as an important regulating factor of aquaculture production efficiency which, also, determines growth and nutrient deposition in fish carcass^3,4^. On the other side, acceptance of farmed fish by the consumer, as a nutritious food, is becoming increasingly debatable since fish encounter multiple stressors in captive conditions and the use of antibiotics in farming yielding an inferior quality farmed fish^5,6^. In future, with high social awareness and health concerned population, there will be a huge demand for the production of superior quality farmed fish, in an energy efficient way. This going to be a major challenge for this fast-growing aquaculture industry.

Compensatory growth (CG) is an accelerated growth response observed in stunted fish seed, the fish has been previously subjected to a stressful condition, under optimal culture conditions^7^. CG is considered as a promising tool to increase aquaculture production and it had been widely experimented in cultivable fish species because of its faster growth rate and enhanced feed utilization^7,8^. In order to induce the CG response, it is essential to keep the fish in catabolic phase, which signals the activation of CG response by adjusting the endogenous energy reserves and endocrine profile^9^. In nature, many fish species undergo a prolonged period of starvation during spawning migration and winter season, so it is acceptable to keep the fish in restricted feeding conditions for prolonged period^10^. Stunting - a high stocking and feed deprivation technique – is practiced by farmers to produce stunted fish that display CG response under optimal culture conditions^11^. In general, larger fish may require extended periods of stunting, to induce the ‘nutritional stress,’ than smaller fish to provoke a CG response^7,12^. Previous studies suggest that in restricted feeding (a stunting phase), fish displayed a significant reduction in body weight gain and survival^12–18^. Many studies found that, under favorable conditions, stunted fish exhibit better growth and feed utilization^16,17,19–22^.

Digestion is a key metabolic process and determines the nutrient availability for all biological functions, including growth, which is controlled by digestive enzyme activity^23–25^. Many authors reported a significant reduction and successful restoration in digestive enzyme activities of fish during restricted and normal feeding conditions, respectively^24,26–31^. In general, fish digestive enzyme activities are affected by the aquaculture feeding practices such as fasting and re-feeding^10^. Hence, profiling of fish digestive enzyme activity, in stunting and post-stunting, is necessary for understanding growth and feed utilization of fish.

The morphometric changes associated with compensatory growth behavior of stunted fish, under optimal culture conditions, affects the nutritional composition and carcass traits of post-stunted fish^8^. In the stunting phase, fish utilize the endogenous energy reserves, lipid and protein, which reduce the carcass nutrient contents and make the stunted fish less nutritious^32^. However, previous studies reported a successful restoration of depleted nutrients in the compensatory growth phase of fish^33–35^. The focus of fish culture, in the world over, has shifted mainly towards on increasing the nutritional quality of farmed fish due to the emerging health concern issues in the contemporary world. In this context, CG can be harnessed for improving the nutritional composition of cultured fish in order to meet the consumer demand^12^.

Among the commercially important tropical marine finfish, milkfish (*Chanos chanos*) is considered as one of the topmost candidate species for marine and brackish water aquaculture, due to its euryhaline nature, meat quality, omnivorous feeding habit, market demand and well-established rearing protocol^36,37^. In late 80’s, an intense effort was made by various researchers to study the effect of stunting in milkfish^38–41^ and therefter not much work had been carried out on stunted milkfish^42^. However, there is a lacuna in understanding the CG response in milkfish. Furthermore, CG response in post-stunting depends on the duration and severity of growth suppression^11^ which also varies among species^22^. So, it is necessary to standardize the optimum duration of stunting for milkfish, in order to maximize the fish production through stocking of stunted seed. Therefore, the present comparative study was conducted to assess the effect of stunting and duration of stunting on the growth, digestive enzymes and carcass quality of milkfish under pond conditions.

## Results

### Water quality parameters

Water quality parameters such as dissolved oxygen (5.0–6.0 mg L^−1^), salinity (10–14 ppt), pH (7.9–8.4), total alkalinity (140–200 mg L^−1^), ammonia (0.02–0.09 mg L^−1^) and nitrate (0.001–0.006 mg L^−1^) did not show much variation during the experimental period and they ideally supported the growth of fish. A slight variation in temperature (25–32 °C) was noted during the experiment due to the onset of winter in the experimental site (during 150 to 180 days and 510 to 540 days of experimental period) (data not shown).

### Growth performance of fish

The initial body weight of fish did not differ significantly between the stunted and normal groups (Table 1). However, at the end of stunting phase, a significant reduction in weight gain and SGR was observed in stunted fish, with respect their normally reared counterparts (control). Overall, the stunted fish reached the body weight of 12.04 ± 0.41 g (T4), 18.05 ± 0.64 g (T8) and 25.43 ± 1.09 g (T12) at the end of stunting phase (Table 2). Similarly, during the process of stunting, increase in stunting duration had negatively affected the survival of fish and a significantly lowered survival rate was recorded in T12 group (51.59 ± 0.21).

**Table 1:** Growth performance and whole body proximate composition (on percentage wet weight basis) observed at the end of stunting phase.

Growth parameters					Proximate parameters				
Treatments		Initial body weight (g)	Final body weight (g)	Survival (%)	Moisture (%)	Dry matter (%)	Protein (%)	Fat (%)	Ash (%)
4 months	Normal	1.20 ± 0.02	107.32^b^ ± 2.96	89.96 ± 0.87	74.76^a^ ± 0.16	25.24^b^ ± 0.16	18.11 ± 0.16	3.41^b^ ± 0.05	3.31 ± 0.07
	Stunted	1.21 ± 0.04	12.04^a^ ± 0.41^1^	61.33 ± 0.12^1^	76.25^b^ ± 0.42^1^	23.75^a^ ± 0.42^2^	17.72 ± 0.14^3^	2.16^a^ ± 0.10^2^	3.57 ± 0.09^1^
8 months	Normal	1.20 ± 0.02	258.24^b^ ± 3.09	89.04 ± 0.69	74.64^a^ ± 0.21	25.36^b^ ± 0.21	18.38^b^ ± 0.06	3.50^b^ ± 0.08	3.12^a^ ± 0.11
	Stunted	1.25 ± 0.03	18.05^a^ ± 0.64^2^	55.92 ± 0.08^2^	77.46^b^ ± 0.51^2^	22.54^a^ ± 0.51^1^	16.19^a^ ± 0.17^2^	2.03^a^ ± 0.05^1^	3.96^b^ ± 0.07^2^
12 months	Norm^a^l	1.20 ± 0.02	367.15^b^ ± 4.29	88.48 ± 0.62	74.30 ± 0.33	26.6 ± 0.33	18.96^b^ ± 0.05	3.74^b^ ± 0.07	3.09^a^ ± 0.05
	Stunted	1.29 ± 0.04	25.43^a^ ± 1.09^3^	51.59 ± 0.21^3^	77.64 ± 0.45^2^	22.36 ± 0.45^1^	14.73^a^ ± 0.09^1^	2.00^a^ ± 0.01^1^	4.40^b^ ± 0.14^3^

Normal fish values calculated from control group at the same culture period of treatment groups. 4 months stunted fish – T4; 8 months stunted fish –T8; 12 months stunted fish – T12.

In each month row, values in the same column with different alphabet superscripts differ significantly (*P* < 0.05). In each parameter, values in the same column with different numerical superscripts differ significantly (*P* < 0.05), where the different duration stunted fish data were compared.

**Table 2:** Growth performance and digestive enzyme activities observed in the present study at different sampling intervals.

Intervals	Pairs	Average body weight gain (g)	Specific growth rate (%/day)	Amylase activity (U/mg protein)	Protease activity (U/mg protein)	Lipase activity (U/mg protein)
At the end of stunting phase	C4	107.32^b^ ± 2.96	1.61^b^ ± 0.064	0.318^b^ ± 0.052	2.62 ± 0.05	6.79^b^ ± 0.42
	T4	12.04^a^ ± 0.41^1^	1.08^a^ ± 0.018^2^	0.198^a^ ± 0.019^2^	2.68 ± 0.10^3^	5.80^a^ ± 0.20^3^
	C8	258.24^b^ ± 3.09	0.45^b^ ± 0.025	0.323^b^ ± 0.019	2.60^b^ ± 0.08	6.29^b^ ± 0.19
	T8	18.05^a^ ± 0.64^2^	0.28^a^ ± 0.009^1^	0.180^a^ ± 0.001^1^	2.06^a^ ± 0.09^2^	4.97^a^ ± 0.11^2^
	C12	367.15^b^ ± 4.29	0.21 ± 0.019	0.328^b^ ± 0.008	2.70^b^ ± 0.05	6.32^b^ ± 0.08
	T12	25.43^a^ ± 1.09^3^	0.22 ± 0.008^1^	0.176^a^ ± 0.020^1^	1.67^a^ ± 0.06^1^	4.20^a^ ± 0.18^3^
At 30^th^ day of post-stunting	C4	145.73^b^ ± 2.09	1.02^a^ ± 0.042	0.395^a^ ± 0.041	2.71 ± 0.05	6.88 ± 0.38
	T4	45.88^a^ ± 1.08^1^	4.46^b^ ± 0.079^2^	0.478^b^ ± 0.017^2^	2.69 ± 0.02^1^	6.75 ± 0.02^2^
	C8	295.49^b^ ± 4.91	0.45^a^ ± 0.031	0.338^a^ ± 0.027	2.64^a^ ± 0.03	6.34^a^ ± 0.03
	T8	80.82^a^ ± 1.28^3^	5.00^b^ ± 0.092^3^	0.585^b^ ± 0.021^3^	5.48^b^ ± 0.13^3^	7.92^b^ ± 0.32^3^
	C12	392.05^b^ ± 4.56	0.22^a^ ± 0.010	0.342^a^ ± 0.011	2.73^a^ ± 0.08	6.32^b^ ± 0.08
	T12	58.61^a^ ± 1.20^2^	2.78^b^ ± 0.078^1^	0.425^b^ ± 0.010^1^	3.28^b^ ± 0.10^2^	5.85^a^ ± 0.05^1^
At 90^th^ day of post-stunting	C4	225.82^b^ ± 3.22	0.52^a^ ± 0.045	0.338 ± 0.021	2.60 ± 0.08	6.51 ± 0.15
	T4	121.97^a^ ± 1.41^1^	1.26^b^ ± 0.054^1^	0.332 ± 0.009^1^	2.62 ± 0.05^1^	6.50 ± 0.08^2^
	C8	344.66^b^ ± 4.09	0.23^a^ ± 0.019	0.313^a^ ± 0.011	2.74^a^ ± 0.02	6.40^a^ ± 0.38
	T8	184.95^a^ ± 3.14^3^	1.12^b^ ± 0.061^1^	0.510^b^ ± 0.005^3^	4.34^b^ ± 0.16^3^	7.55^b^ ± 0.23^3^
	C12	457.25^b^ ± 5.02	0.32^a^ ± 0.012	0.387 ± 0.006	2.81 ± 0.38	6.36 ± 0.38
	T12	144.64^a^ ± 2.91^2^	1.39^b^ ± 0.059^2^	0.390 ± 0.005^2^	2.84 ± 0.11^2^	6.04 ± 0.19^1^
At the end of post-stunting phase	C4	547.14^b^ ± 5.94	0.17 ± 0.005	0.324 ± 0.016	3.05 ± 0.29	5.81 ± 0.18
	T4	477.57^a^ ± 6.64^2^	0.17 ± 0.006^1^	0.351 ± 0.029^2^	2.95 ± 0.04^2^	5.85 ± 0.11
	C8	547.14 ± 5.94	0.17^a^ ± 0.005	0.324^a^ ± 0.006	3.05 ± 0.29	5.81 ± 0.18
	T8	537.60 ± 5.29^3^	0.38^b^ ± 0.012^2^	0.359^b^ ± 0.011^2^	3.23 ± 0.05^3^	6.08 ± 0.12
	C12	547.14^b^ ± 5.94	0.17^a^ ± 0.005	0.324 ± 0.006	3.05^b^ ± 0.29	5.81 ± 0.18
	T12	251.05^a^ ± 5.47^1^	0.43^b^ ± 0.016^2^	0.325 ± 0.013^1^	2.53^a^ ± 0.02^1^	5.94 ± 0.29

T4-4 months stunted fish; T8-8 months stunted fish; T12- 12 months stunted fish; C4, C8, C12 represents normal fish data collected from control group (C) at different time intervals in respect to treatment group. In each sampling interval, values in the same row with different alphabet superscripts differ significantly (*P* < 0.05). In each sampling interval, values of T4, T8 & T12 with different numerical superscripts differ significantly (*P* < 0.05), where the different duration stunted fish data were compared.

Statistical analysis performed at different intervals found that stunting and duration of stunting had significantly affected the body weight gain and specific growth rate of milkfish in stunting and post-stunting phases. In the initial phase of post-stunting (30^th^ day), it was found that fish reared in T8 group exhibited a significantly higher body weight gain and SGR values (80.82 ± 1.28 g & 5.00 ± 0.092) as compared to T4 (58.61 ± 1.20 g & 4.46 ± 0.079) and T12 (45.88 ± 1.08 g & 2.78 ± 0.078) (Table 2). A similar pattern was exhibited in the 90^th^ day of the study. However, the study did not find any significant difference in body weight of control and T8 groups at the end of post-stunting (Table 3). Further, the study found a significantly higher net production in T8 (49.13 ± 1.03 Kg) and control (47.97 ± 1.55 Kg) groups.

**Table 3:** Growth performance and whole body proximate composition (on percentage wet weight basis) observed at the end of post-stunting phase.

Treatments	Growth parameters						Proximate parameters				
	Average body Weight Gain (g)	Net production (Kg)	AFCR	FER	PER	Survival (%)	Moisture (%)	DM (%)	Protein (%)	Fat (%)	Ash (%)
C	545.94^d^ ± 6.02	47.97^c^ ± 1.55	3.75^c^ ± 0.02	0.27^a^ ± 0.001	1.54^a^ ± 0.03	87.67^a^ ± 0.76	74.55^a^ ± 0.34	25.45^b^ ± 0.34	17.75^a^ ± 0.17	3.34 ± 0.10	3.23 ± 0.12
T4	465.53^b^ ± 5.05	42.11^b^ ± 2.12	3.30^b^ ± 0.05	0.30^b^ ± 0.006	1.75^b^ ± 0.02	88.17^a^ ± 1.12	72.75^b^ ± 0.71	27.25^a^ ± 0.71	20.66^b^ ± 0.42	3.12 ± 0.19	3.09 ± 0.02
T8	519.55^c^ ± 8.82	49.13^c^ ± 1.03	3.03^a^ ± 0.04	0.33^c^ ± 0.002	1.91^c^ ± 0.03	91.38^b^ ± 0.07	71.51^b^ ± 0.53	28.49^a^ ± 0.53	21.91^c^ ± 0.51	3.10 ± 0.27	2.98 ± 0.20
T12	225.62^a^ ± 4.31	21.52^a^ ± 1.91	3.28^b^ ± 0.05	0.30^b^ ± 0.005	1.76^b^ ± 0.01	85.71^a^ ± 2.16	72.73^b^ ± 0.50	27.27^a^ ± 0.50	19.74^b^ ± 0.35	3.24 ± 0.08	3.28 ± 0.24

C – normal fish; T4-4 months stunted fish; T8-8 months stunted fish; T12- 12 months stunted fish.

Values in the same column with different superscripts differ significantly (*P* < 0.05) for each parameter.

Fish reared normally (control) displayed an increased AFCR (3.75 ± 0.02) and lower FER and PER values (0.27 ± 0.01 & 1.54 ± 0.03). Among the stunted groups, fish reared in T8 group displayed a better efficacy in feed utilization in terms of lower AFCR (3.03 ± 0.04) and higher FER and PER values (0.33 ± 0.01 & 1.91 ± 0.03) at the end of post-stunting phase. Similarly, significantly higher survival rate (91.38 ± 0.07) was observed in T8 group at the end of post-stunting (Table 3).

### Digestive enzyme assays

A significant reduction in digestive enzyme activities (except protease activity in T4 group) was recorded in stunted fish at the end of stunting phase when compared with their respective control (Table 2). At the end of stunting phase, the study found that protease activity was significantly reduced (among the digestive enzymes) with increase in the stunting duration and a significantly lowered protease activity (1.67 ± 0.06 U/mg protein) was recorded in T12.

**Figure 1:**
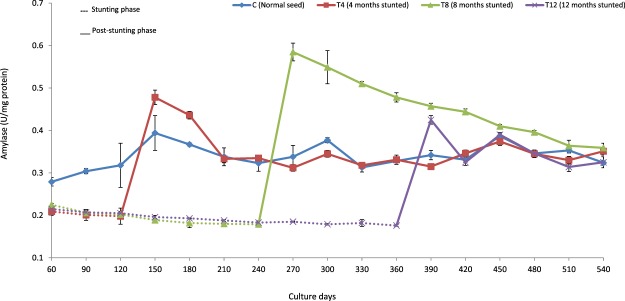
Amylase activity (U/mg protein) of different duration stunted and normal milkfish observed at 30 days interval during stunting and post-stunting phases.

**Figure 2:**
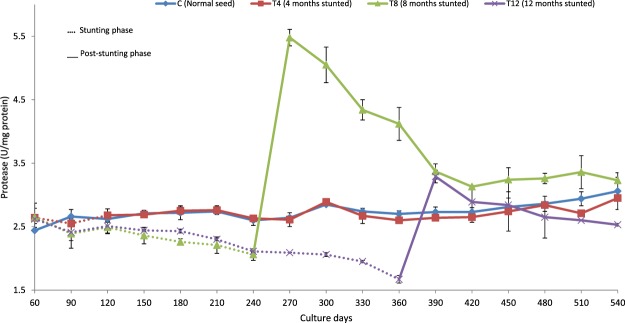
Protease activity (U/mg protein) of different duration stunted and normal milkfish observed at 30 days interval during stunting and post-stunting phases.

**Figure 3:**
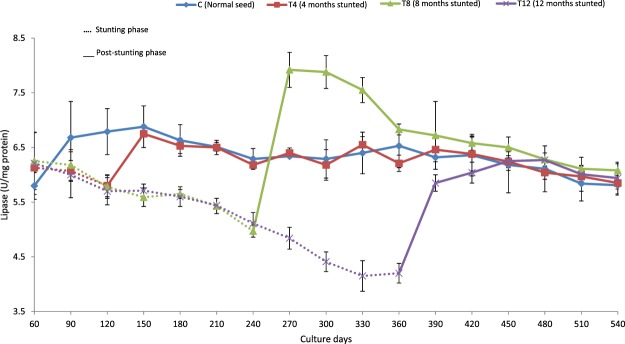
Lipase activity (U/mg protein) of different duration stunted and normal milkfish observed at 30 days interval during stunting and post-stunting phases.

The study found that there was no alteration in lipase and protease activities of T4 group due to stunting. However, stunting had negatively affected the lipase activity of T12 group in the initial phase of post-stunting. Fish stunted for 8 months (T8) displayed significantly higher digestive enzyme activities for an extended period in post-stunting phase. On the 90^th^ day of post-stunting, significantly higher levels of amylase, protease and lipase (0.510 ± 0.005, 4.34 ± 0.16 & 7.55 ± 0.23 U/mg protein) were recorded in T8 group (Figs 1–3). At the end of post-stunting phase, no significant differences were evident in lipase activity but T12 and T8 groups recorded significantly lower and higher protease activity, respectively (Table 2).

### Proximate composition of fish

Stunted fish displayed significantly higher moisture and lower dry matter contents at the end of stunting phase when compared with their respective control fish (Table 1). The T12 group exhibited significantly lower protein and fat contents (14.73 ± 0.09% & 2.00 ± 0.01%) at the end of stunting phase. At the end of post-stunting phase, lower moisture and higher dry matter contents were recorded in post-stunted fish. Among the stunted group, significantly higher protein content (21.91 ± 0.51%) was obtained in T8 group (Table 3). No significant differences were observed in fat and ash contents between the different treatments.

### Texture and carcass trait analysis

The study did not find any significant difference in fillet texture of milkfish at the end of post-stunting (Table 4). The hardness of fillet was in the range of 34.64 to 39.78 N. Fillet cohesiveness and elasticity were in the range of 3.34 to 4.52 and 1.59 to 2.41 mm, respectively. Fish reared under normal conditions (control group) and the T12 group showed significantly lowered carcass trait yields such as dressed (83.33% & 82.30%) and headless dressed (62.94% & 61.60%) (Table 4). However, no significant differences were found in dressed carcass yields between T8 and T12 group. Further, the study found significantly higher meat (49.30% & 47.10%) and lower bone contents (5.56% & 5.68%) in T8 and T12 groups, respectively.

**Table 4:** Carcass traits and texture quality of milkfish at the end of post-stunting phase.

Dressed carcass traits	C	T4	T8	T12
Fresh body weight (g)	547.14 ± 5.94	477.57 ± 6.64	537.60 ± 5.29	251.05 ± 5.47
Dressed body weight (g)	455.93 ± 3.61	403.74 ± 4.56	457.75 ± 3.40	206.64 ± 3.14
Dressed percentage (%)	83.33^ab^ ± 0.35	84.54^b^ ± 0.76	85.12^b^ ± 0.20	82.30^a^ ± 0.73
Headless dressed weight (g)	344.30 ± 1.01	316.24 ± 2.21	361.37 ± 2.49	154.66 ± 1.78
Headless dressed percentage (%)	62.94^a^ ± 0.60	66.23^b^ ± 0.43	67.20^b^ ± 0.51	61.60^a^ ± 0.64
Meat yield (g)	213.80 ± 1.75	190.16 ± 1.23	225.69 ± 1.92	84.19^b^ ± 1.54
Meat (%)	46.89^b^ ± 0.71	47.10^b^ ± 0.28	49.30^c^ ± 0.84	40.74^a^ ± 2.01
Bone yield (g)	33.83 ± 0.56	22.94 ± 0.37	25.45 ± 0.30	12.56 ± 0.23
Bone (%)	7.42^c^ ± 0.52	5.68^a^ ± 0.15	5.56^a^ ± 0.24	6.08^b^ ± 0.31
Hardness (N)	34.64 ± 4.49	38.12 ± 4.66	39.78 ± 2.95	38.68 ± 3.57
Cohesiveness (ratio)	3.34 ± 0.63	4.22 ± 0.56	4.52 ± 0.45	3.27 ± 0.14
Elasticity (mm)	1.81 ± 0.34	2.01 ± 0.38	2.41 ± 0.39	1.59 ± 0.67

C – normal fish; T4-4 months stunted fish; T8-8 months stunted fish; T12- 12 months stunted fish.

Values in the same row with different superscripts differ significantly (*P* < 0.05) for each parameter.

## Discussion

### Stunting phase

The present study found a significant reduction in body weight gain and specific growth rate of fish at the end of stunting phase. A significant reduction in body weight gain and SGR was reported in *Oncorhynchus mykiss*^10^, *Pelteobagrus fulvidraco*^17^, *Argyrosomus regius*^18^, *Leiocassis longirostris*^35^, *Hippoglossus hippoglossus*^43^, and *Sparus aurata*^44^ in feed restriction or starvation or stunting conditions. African catfish exposed to 2 months of complete feed restriction showed significantly reduced body weight gain^13^. A contrary result was reported in Gibel carp where short-term feed restriction (1 & 2 weeks) did not reduce the body weight^34^. However, in a supportive finding, significant reduction in body weight was reported in long-term starved (80 days) channel catfish^45^. In general, food type and ration size largely affect the fish growth^46^. So the sub-optimal feeding regime followed in stunting phase is the reason behind lower body weight gain of stunted fish compared to their counterpart, normally fed fish.

In fish, the early phase of nutritional imbalances negatively affects its growth and survival^47^. Agreeing with this, the present study found a significantly reduced survival rate with increased duration of stunting. Previous finding also reported a similar survival rate in prolonged stunting of milkfish^40^. However, a better survival was evident in T8 and T12 groups. A comparable result was reported in *Hoplias malabaricus* where lower metabolic rate allowed the fish to survive for a prolonged period (180 days) of feed restriction^48^. Further, short-term starvation might induce metabolic changes or convinces the fish to reduce its metabolic rate or energy expenditure (e.g. reduction in locomotion) in order to increase chances of survival but prolonged starvation may ultimately cause death^5,7,49^.

Stunted fish exhibited reduced digestive enzyme activities when compared with the control group at the end of stunting phase, except for protease activity in T4 group. In Atlantic cod, short-term starvation (25 days) did not affect protease activity^50^. On the contrary, short-term starvation in *Labeo rohita* decreased the activity of amylase, protease and lipase^31^. A comparable result reported in trout, showed decreased digestive enzyme secretion during prolonged starvation^51^. The reason for reduction in digestive enzyme activity in T8 and T12 groups, therefore, may be attributed to prolonged duration of stunting. In prolonged starvation, atrophy of gut tissues was reported in salmon^52^, bluegill sunfish^53^ and brown trout^54^ which probably was the reason why digestive enzyme activities were affected. Additionally, chronic starvation results in the reduction of the pyloric caeca, intestinal microvilli, length of intestine and diameter of the intestine^53,55^, which also directly affects digestive enzyme activities. In the present study, amylase activity did not get reduced with the increase in stunting duration. The possible explanation for this is the feed used in the present study, de-oiled rice bran with 66% carbohydrate, since fish used to adjust its digestive enzyme activity based on its diet^56^.

Animals undergo hibernation, a state of reduced metabolic activity, during unfavourable environmental conditions, which allows them to use their stored energy reserves^57^. Stunted fish in the present study exhibited a higher amount of moisture when compared with the control group at the end of stunting phase. Contrary to this, the moisture content of golden perch did not change in prolonged feed restriction of 210 days^33^. However, African catfish in short-term starvation (66 days) displayed significantly increased moisture content^13^. Fasted fish has been reported to exhibit higher moisture content due to tissue hydration, high rate of water absorption and utilization of reserved nutrients (fat & protein) for metabolic activities^32,58^. In the present study, a significant reduction in fat content was observed in the initial phase of stunting in milkfish. Short-term starvation in Plaice significantly reduced the fat content^59^. Also, starvation in rainbow trout significantly reduced the fat and increased the moisture contents^60^. In fish, lipids are the main source of energy which is broken down early in the fasting phase^7^. However, significantly lower protein content was recorded in T8 and T12 groups at the end of stunting phase. In prolonged stunting, fish use protein as an energy source, via gluconeogenesis^61^. In four-week feed deprivation, hybrid tilapia (*O. mossambicus* × *O. niloticus*) exhibited significantly lower carcass protein content than the other groups (one, two and three-week feed deprived)^62^. A significant reduction in protein content was reported in starved *Clarius gariepinus* and *Oreochromis mossambicus*^13,63^.

### Post-stunting phase

The weight lost during stunting could be compensated in re-feeding phase partly or fully by fish. However, the degree of compensation in re-alimentation phase depends on the duration and severity of food restriction imposed in stunting phase^64^. In the present study, T8 group exhibited complete growth compensation (the body weight of T8 group did not differ significantly from the control group at the end of the post-stunting) and T4 and T12 groups displayed partial growth compensation (where a rapid restoration of body weight gain was observed in the initial phase of re-alimentation but did not restore fully as that of the control group) during the re-alimentation period. Duration of food restriction in stunting phase, to induce nutritional stress, varies among species to induce CG response in post-stunting. In Arctic charr, 3 weeks of deprived feeding was insufficient to induce compensatory growth whereas the same fish in 6 months of cyclic restricted feeding exhibited complete growth compensation^12,64^. In fish, feed restriction should reach its threshold limit in stunting phase, in order to induce compensatory growth in post-stunting^25^. Lower body weight gain and SGR were observed in T12 group which indicates that prolonged stunting negatively affects the growth of milkfish. Similar to this, stunting of rohu for more than 6 months negatively affects its growth performance in the grow-out phase^65^.

CG is characterized by an elevated SGR and improved feed conversion efficiency in the re-feeding stage^7,9^. The present study found an increased SGR, in the initial phase of post-stunting, and better FCR in stunted fish, which confirms their compensatory behaviour in re-feeding phase. Nile tilapia in post-stunting phase exhibited better growth and feed utilization in terms of higher SGR, FCE and PER and lower FCR than the continuously fed fish^21^. Feed restricted Pacu, *Piractus brachypomus*, expressed better FCR and higher FE and PER than the control group^19^. The FCR value of the present study was in the range of reported FCR value (3.9:1) of extensive milkfish culture, using rice bran^66^. Among the stunted groups, T8 group exhibited better FCR (3.03). It can be further correlated with the better specific growth rate (5.00%/day) of T8 group, in the initial stage of re-feeding phase. Improved feed utilization in post-stunting phase could be identified by its better growth rate^26^.

Elevated digestive enzyme activity was observed in stunted fish, compared with the control group, in the post-stunting phase. Rapid restoration of an atrophied intestine was reported in re-feeding phase^24^. *Colossoma macropomum*, subjected to 7-weeks of feed starvation in the re-feeding stage showed a drastic increase in amylase activity^26^. The increased digestive enzyme activity in re-alimentation phase was due to the increased availability of feed, after a food restriction period^30^. In rainbow trout, significantly reduced lipase activity reported during starvation phase, however, the reduced activity was successfully restored in re-feeding phase^27^. The feed restricted tongue sole (*Cynoglossus semilaevis*) displayed a significantly higher lipase activity than the normally fed fish in the re-feeding stage^29^. In fish, faster growth rate used to be accompanied by elevated digestive enzyme activities which improve digestive capacity^25^. In *Labeo rohita*, an increased amylase and protease activities were attributed to compensatory growth^67^. In the present study, the elevated digestive enzyme activities of stunted fish during post-stunting phase further confirmed the compensatory growth response of stunted fish.

The availability of more feed in re-feeding phase supplies more quantity of nutrients to intestinal lumen for tissue regeneration of stunted fish^10^. However, the magnitude of tissue regeneration depends on the threshold point that the fish experience in stunting phase. The study did not find a significant difference in digestive enzyme activities at 90^th^ day of post-stunting, among T4, T12 and control groups, indicating that stunting of milkfish for 4 and 12 months did not produce any positive impact on digestive enzymes. However, significantly higher digestive enzyme activity was observed in T8 group which can be further correlated with their efficient feed utilization (better FCR and improved FCE & PER) and complete growth compensation.

Stunted fish in re-feeding phase display compensatory growth behaviour which restores the depleted nutrients or body composition^8^. In the present study, stunted fish successfully restored their depleted nutrients at the end of post-stunting phase. In compensatory growth phase, fish tends to aggregate a higher amount of protein in tissues^68^. The present study found a higher amount of crude protein in stunted fish. A significantly higher crude protein was reported in post-stunted Giebel carp than the normally reared fish^34^. Among the stunted groups, T8 group exhibited a significantly higher amount of protein content. The nutrient accretion of post-stunted fish used to vary among species which is mainly influenced by re-feeding schedule^69^. In fish, the increase in nutrients (protein and lipid) decreases the ash content, which comes from a non-edible portion of fish such as the scale and bones^70^. This ash content was well within the range reported in previous studies on milkfish^71,72^. The nutritional quality of the stunted fish is grossly influenced not only by re-feeding schedule, but also by the duration of stunting and optimized stunting duration (e.g. 8 months) produces a fish with superior nutritional quality.

### Carcass traits and texture quality

Most commonly assessed flesh quality parameter is texture, using an instrument called texturometer. The study did not find any significant difference in fillet texture quality among the treatments. A similar finding was earlier reported in turbot, where adult turbot exhibiting compensatory growth response did not show any significant variation in its fillet texture^73^. Variation in nutritional state of fish, especially in fat content, influences the taste and texture quality of fish muscle^74^.

The present study found a slightly higher dressed output in T8 (67.20%). The final dressed output of farmed fish normally differs, depending on species, and in general, it is reported to be around 60%^75^. In fish, the final dressed out-put is affected by the weight of the head, visceral, scale and fins^75^. The compensatory growth pattern of post-stunted fish tends to deposit more lean body mass (accumulate more energy reserves and nutrients)^12,33^ and distribute lower energy for the development of body parts^8^ which might have contributed for the increased final dressed output of post-stunted fish. Previously, also, a variation in muscle distribution has been reported in fish^76^, which may be correlated to the difference in meat and bone contents in the present study. The significantly lower bone percentage recorded in post-stunted fish can be further explained through the proven fact that stunted fish tends to accumulate the muscle mass in post-stunting phase^12,75,77^.

## Conclusion

This study found that rearing of stunted milkfish over normal milkfish has advantages in terms of improved growth performance and delivers a better quality product. However, stunting of milkfish for 4 months was insufficient and 12 months was excessive to favor the positive compensatory growth response. So, the optimum stunting duration for stunted milkfish seed production is 8 months, to improve the overall production of milkfish with a nutrionally better product, which, also, help the stakeholders to overcome the contemporary challenges in milkfish culture. Further, the changes in stunted fish during post-stunting phase at physiological level has been poorly understood, especially immunological responses. Therefore, a challenge study using histological and molecular tools will be a welcome to understand more about the immunity and physiological recovery of stunted fish in post-stunting phase.

## Materials and Methods

### Experimental fish and rearing conditions

All the methods used in the present study followed relevant guidelines and regulations. Also, the competent authority (Indian Council of Agricultural Research-Central Institute of Fisheries Education) approved the experiment and protocols of the present study.

Milkfish fry (average weight of 0.58 ± 0.02 g and length of 4.38 ± 0.05 cm) collected from the wild were acclimatized to captive pond conditions for 30 days at the experimental site, brackish water fish farm of Central Institute of Fisheries Education (CIFE), Andhra Pradesh, India. The experiment followed a completely randomized design with one control (C - normal seed) and three treatment groups (T4–4 months, T8 - 8 months and T12 - 12 months stunted seed), in triplicates. The whole experiment was carried out for 18 months in earthen ponds of 0.02 ha (200 m^2^) with a length and breadth of 20 m × 10 m. Prior to stocking, water in the experimental ponds were completely drained and the ponds were sun dried for 10 days. Then, the water, collected from creek inlet canal of farm, has been filled and disinfected using commercial bleach (Ca(ClO)_2_) at a dose of 20 Kg/ pond. The pond water has been left as such for 10 days and then it was stocked with fish seed, after confirming there is no trace of chlorine left in the pond water.

In stunting phase, the fish, acclimatized for 30 days (average weight of 1.25 ± 0.03 g and length of 5.53 ± 0.03 cm), were stocked at the rate of 0.2 million fry/ha and fed at sub-optimal level, 1% of body weight^39,41^. Each treatment was stocked separately, in triplicates, and reared for different durations as per their treatment stunting duration. The control group were stocked separately, in triplicates, and reared under extensive culture condition (stocking density - 5000 numbers/ha; feed - de-oiled rice bran and fed twice a day for 3% of body weight) for 18 months. The post-stunting phase commenced, immediately, once the respective stunting duration has completed. In post-stunting phase, the stocking density of stunted juvenile was adjusted (5000 numbers/ha) and fed with an optimum level (3%) of rice bran for different durations to complete the total experimental period of 18 months. The study followed a subsequent rearing period *viz*., control (18 months normal rearing), T4 (4 months stunting: 14 months of post-stunting), T8 (8 months stunting: 10 months of post-stunting) and T12 (12 months stunting: 6 months of post-stunting). Water quality parameters such as temperature, dissolved oxygen, salinity, pH, total alkalinity, ammonia and nitrate were monitored regularly following the standard procedures^78^.

### Growth performance analysis

Sampling was carried out at monthly intervals in both phases and each time 50 fish/pond were sampled to record the total weight and length of the individual fish using a standard scale and weighing balance having a precision of 1 mm and 0.1 g, respectively. Growth parameters such as Specific Growth Rate (SGR, %/day-1) = [(ln Final weight − ln Initial weight)/Number of days] × 100; Net production (Kg) = average weight of individual fish × number of fish harvested; Apparent Feed Conversion Ratio (AFCR) = Feed given (dry weight)/Body weight gain (wet weight); Feed Efficiency Ratio (FER) = Body weight gain (wet weight)/Feed given (dry weight); Protein Efficiency Ratio (PER) = Body weight gain (wet weight)/ Crude Protein in feed and Survival (%) = (Total number of fish harvested/Total number stocked) × 100, were estimated as per standard formulas.

### Tissue collection and digestive enzyme assays

The intestinal samples were collected from each treatment (n = 9/sampling) at various intervals (end of both phases and 30^th^ & 90^th^ day of post-stunting) to test the stunting and compensatory effect on digestive enzymes. For this, 20% of intestinal tissue homogenate was prepared using a 0.25 M chilled sucrose solution. The tissue solution was homogenized thereafter, using a tissue homogenizer and centrifuged at 8000 rpm for 10 minutes. Finally, the supernatant was collected and used for further analysis.

The amylase activity of intestinal samples was determined using the DNS method^79^. The reducing sugars produced due to the action of glucoamylase and α-amylase on carbohydrate was estimated using 3,5-dinitrosalicylic acid (DNS) method. The reaction mixture consisted of 1% (w/v) starch solution, 0.1 M phosphate buffer (pH 7.0) and the tissue homogenate. The reaction mixture was incubated at 37 °C for 30 min. Then DNS was added and kept in a water bath for 5 min. After cooling, the reaction mixture was diluted with distilled water and absorbance was measured at 540 nm in a UV spectrophotometer. One unit of amylase activity was defined as the number of moles of maltose released from starch per minute per milligram of protein.

Protease activity of intestinal samples was determined by the casein digestion method^80^. The reaction mixture consisted of 1% casein in 0.05 M trisphosphate buffer (pH 7.8) and tissue homogenate. Then the mixture incubated for 10 min at 37 °C. After ten minutes, the reaction was stopped by the addition of 10% TCA and the whole content was filtered. Then the absorbance was measured at 280 nm in a UV –VIS spectrophotometer (Thermo Scientific, Genesys, 10S UV-VIS). The protease activity was determined from the tyrosine standard curve and expressed as micromole of tyrosine released min^−1^ mg^−1^ protein.

Lipase activity of intestinal samples was determined based on the titrimetric method^81^. The reaction mixture consists of distilled water, tissue homogenate, 0.1 M phosphate buffer (pH 7.0) and olive oil emulsion. The mixture was shaken well and incubated at 27 °C for 24 h. Then, 95% alcohol and two drops of phenolphthalein indicator were added and titrated against 0.05 N NaOH until the appearance of permanent pink colour. One unit of lipase activity was considered as the number of micromoles of fatty acids released per minute per milligram of protein.

### Analysis of proximate composition, fillet texture, and carcass quality

At the end of stunting and post-stunting phases, fish samples were collected (n = 10/treatment) and their whole body nutrient content were analyzed. The moisture content of the sample was measured by drying the pre-weighed sample in the hot air oven at 105 ± 5 °C for 18–24 hrs^82^. Then, the dried samples were homogenized and used for further analysis. The values obtained were converted and expressed in wet weight basis. The lipid, protein and ash contents were estimated by Soxhlet, Kjeldahl and dry weight (using muffle furnace) methods, respectively^82^.

The textural characteristics of post-stunted fish at the end of post-stunting phase were measured using a texture analyser (Perten, TVT-300XP(H), Perten Instruments AB, Sweden) using a 20 mm cylindrical probe which was maintained at 5 mm distance with a trigger force of 50 N. The initial speed and test speed was set at 1 mm/s. The sample height and starting distance from the sample were set at 5 mm and 3.8 mm, respectively. A two-bite test was conducted to assess the elasticity, cohesiveness and hardness of the fillets. Five measurements were performed on each sample size of 5 × 5 cm (diameter × length).

The collected fish (n = 10/treatment) were dissected (after evisceration or removal of head and fins) and dressed to study carcass traits. The following carcass traits were evaluated using a standard technique^83^, as per the formulas, Dressed percentage = weight of dressed body/total body weight × 100; Headless dressed percentage = weight of headless dressed body/total body weight × 100; Meat yield (%) = weight of meat/dressed body weight × 100; Bone yield (%) = weight of bone/dressed body weight × 100.

### Statistical analysis

The collected data of growth and carcass quality trait parameters were analyzed by one-way analysis of variance (ANOVA) using SPSS 20.0 version. Duncan’s multiple range test was used for post hoc comparison of means and data is presented as mean ± S.E. Further, to test the effect of stunting and the duration of stunting on growth and digestive enzymes, the data collected at different intervals were subjected to repeated measures analysis of variance (RM-ANOVA). The statistical difference between stunted and normal groups fish was estimated using student’s t-test. Statistical significance for all the analysis was set at P < 0.05.
